# Reducing delays in radiotherapy initiation post CT simulation: a quality improvement study from a rural cancer center in India

**DOI:** 10.3332/ecancer.2026.2116

**Published:** 2026-05-01

**Authors:** Pragyat Thakur, Nagarjun Ballari, Anureet Kaur, Tapas Kumar Dora, I Vedamanasa, Arshdeep Kaur, Ashish Gulia

**Affiliations:** 1 Department of Radiation Oncology, Homi Bhabha Cancer Hospital and Research Centre (HBCHRC), Tata Memorial Centre, Affiliated to Homi Bhabha National Institute (HBNI), Mullanpur, Punjab, India; 2 Department of Radiation Oncology, Homi Bhabha Cancer Hospital (HBCH), Tata Memorial Centre, Affiliated to Baba Farid University of Health Sciences (BFUHS), Sangrur, Punjab, India; 3 Department of Surgical Oncology, Homi Bhabha Cancer Hospital and Research Centre (HBCHRC), Tata Memorial Center, Affiliated to Homi Bhabha National Institute (HBNI), Mullanpur, Punjab, India; a https://orcid.org/0009-0008-6823-4395

**Keywords:** radiotherapy, quality improvement, CT simulation, time to treatment, workflow redesign, root cause analysis, treatment delay

## Abstract

Radiation therapy (RT) is a cornerstone in the treatment of solid tumours, with more than half of all cancer patients requiring it for curative or palliative intent. However, delays in initiating RT after CT simulation (CT sim) can significantly impact clinical outcomes by increasing recurrence risk, triggering re-simulation due to anatomical shifts and causing psychological and logistical distress for patients and caregivers. This prospective quality improvement (QI) study was conducted at a rural cancer center in India from April to December 2022, aiming to reduce the time from CT sim to RT initiation. Using the A3 methodology in collaboration with Enable Quality, Improve Patient Care India, Stanford Medicine and the National Cancer Grid, a root cause analysis was conducted, followed by key driver identification via Pareto analysis. A series of Plan-Do-Study-Act (PDSA) cycles led to targeted interventions, including written standard operating procedures (SOPs) for scheduling, standardised patient instructions, clearly defined staff roles and stakeholder education. Data from 200 patients planned for radical treatment were analysed weekly, with treatment timelines plotted on a run chart. At baseline, the median delay from simulation to treatment initiation was 18 days. After implementation of interventions in July 2022, this was reduced to 12 days by September and further to 10 days by November, a 44.4% reduction . Additionally, the re-simulation rate dropped from 10% to less than 1%. No patient experienced a delay beyond the prescribed date, and importantly, staff feedback confirmed that the revised workflow did not increase perceived workload. The interventions were institutionalised through SOPs and monitored via real time dashboards, ensuring sustainability into 2024. This study demonstrates that targeted, system-level interventions developed using accessible QI methodologies can lead to meaningful reductions in RT delays and operational inefficiencies in low-resource environments. The model is feasible, cost-effective and adaptable to other cancer centers aiming to optimise timely RT delivery and improve patient outcomes.

## Introduction

Radiation therapy (RT) is a cornerstone in the management of solid tumours, with over half of all cancer patients receiving it at some point during their treatment course, either alone or in combination with other modalities [1–3]. The timely initiation of RT is critical for optimising clinical outcomes, especially in settings where treatment delays are common.

Time to treatment initiation (TTI), the interval between diagnosis and the start of therapy, is a well-established determinant of cancer-related outcomes [4]. In head and neck squamous cell carcinoma (HNSCC), timely postoperative RT is essential for achieving locoregional control and improving survival, particularly in patients with advanced stage or high-risk disease [5, 6]. Delays in initiating RT have been consistently linked to higher recurrence rates [7, 8] and increased cancer specific and overall mortality [9–11]. Beyond oncologic outcomes, delays may also lead to increased rates of resimulation due to changes in patient anatomy, increased psychological distress and place a greater burden on healthcare systems [12].

Despite significant advancements in RT technology, delays between CT simulation (CT Sim) and treatment initiation remain a persistent challenge, particularly in low-resource or rural healthcare settings. While existing literature has largely focused on delays from diagnosis to treatment [13] or postoperative RT timing, the specific interval between CT sim and the delivery of the first RT fraction remains understudied.

This quality improvement (QI) study was conducted at a rural cancer center in India with the objective of identifying key factors contributing to post simulation delays in RT initiation. Using structured QI tools, we aimed to implement targeted interventions to reduce the delay between CT sim and initiation of RT for cancer patients across various tumour sites .

## Materials and methods

The study spanned a 9 month period, from April to December 2022, and enrolled a total of 200 patients who underwent CT sim for planned external beam RT.

The QI initiative followed the A3 methodology, a structured problem-solving approach widely used in healthcare process optimisation. It was implemented in collaboration with the Enable Quality, Improve Patient Care (EQUIP India) program of Stanford Medicine and the National Cancer Grid, India. The A3 process involved defining the problem, analysing the root causes, implementing targeted interventions and assessing outcomes.

**Figure 1.**
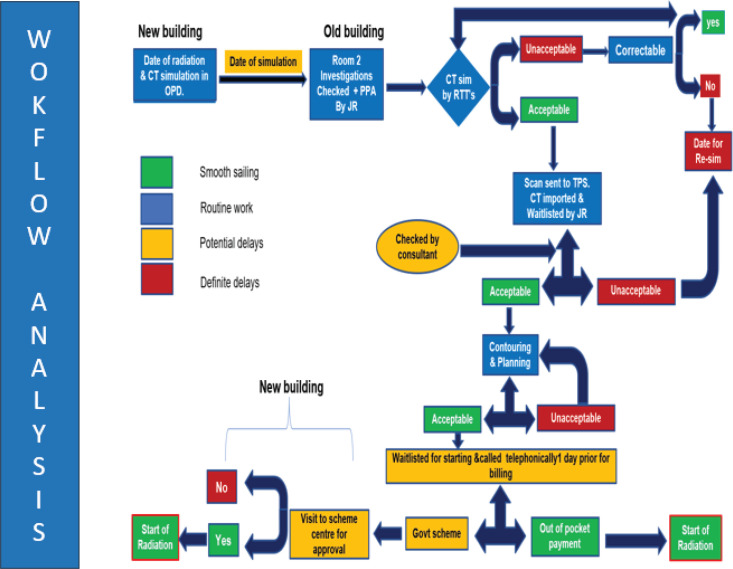
CT sim to RT workflow derived from Gemba Walk analysis. This figure illustrates the end-to-end workflow from CT sim to the initiation of RT at our center, as mapped during a Gemba Walk exercise. Key steps, such as simulation, planning and scheduling, are highlighted along with critical transition points and bottlenecks contributing to delays. Abbreviations: CT, Computed Tomography; JR, Junior Resident; OPD, Outpatient Department; PPA, Pre-Planning Audit; Re-sim, Resimulation; RTT, Radiation Therapy Technologist; Sim, Simulation; TPS, Treatment Planning System.

A Gemba Walk, an observational process in which team members visit the clinical workspace to directly observe workflows (Figure 1), was conducted during regular departmental hours. This helped identify systemic and process-level inefficiencies contributing to delays in treatment initiation.

A root cause analysis (RCA) was subsequently performed, involving multidisciplinary stakeholders including radiation oncologists, medical physicists, radiotherapy technologists (RTTs), nursing and administrative staff. An EQUIP questionnaire (Table 1) was specifically developed to assess various contributing factors to RT initiation delays using a standardised five point Likert scale (Table 2), allowing respondents to rate their level of agreement with each identified cause. The questionnaire was developed through an iterative, consensus-driven process involving frontline stakeholders, including radiation oncologists, medical physicists and RTTs with direct experience in workflow bottlenecks. Initial item generation was informed by insights from Gemba walks, fishbone analysis and literature on radiation workflow delays. To ensure content validity, the draft questionnaire was reviewed in two structured feedback sessions by a panel of five senior faculty members with expertise in radiation oncology operations and QI. Items were revised based on clarity, relevance and redundancy. A pilot administration was then conducted among a representative sample of ten staff members across different roles (RTTs, residents and nursing staff) to assess response variability and comprehension. Internal consistency was evaluated using Cronbach’s alpha, which demonstrated acceptable reliability (α = 0.81). The final version focused on identifying specific factors delaying RT initiation post CT sim.

A fishbone diagram (Ishikawa analysis) was constructed to categorise contributing factors into major domains. Pareto analysis, based on the 80/20 principle, which posits that approximately 80% of delays can be attributed to 20% of the underlying causes, was employed to prioritise high-impact drivers of delay according to their frequency and relative contribution. Informed by the RCA and Pareto analysis, a multidisciplinary team developed Targeted interventions using the Plan-Do-Study-Act (PDSA) framework. Interventions were tested iteratively over several cycles to evaluate feasibility, scalability and effectiveness.

The primary outcome defined for this QI initiative was the proportion of patients scheduled to initiate RT within 10 days of CT sim, based on the institutional benchmark for timely treatment initiation.

The secondary outcomes included predefined process measures:

1. the rate of re-simulations,
2. patient compliance with scheduled appointments,
3. patient satisfaction with the workflow and
4. the degree of workflow redundancy and administrative load on staff.

To evaluate these outcomes, weekly CT Sim to RT initiation intervals were prospectively recorded throughout the study period. These data were plotted on run charts as part of routine QI monitoring. Interventions implemented during the PDSA cycles were annotated directly onto the charts to allow temporal association between process changes and subsequent variations in outcome measures.

Following the intervention phase, a structured postimplementation monitoring plan was introduced in January 2023. This included weekly audit rounds, integration of key performance indicators (e.g., CT Simtotreatment interval, re-simulation rate) into departmental dashboards and structured orientation of incoming staff to the revised Standard Operating Procedures (SOPs). These activities were integrated into routine departmental operations and continued through 2024 to promote sustainability and institutionalisation of the intervention bundle.

Data collection and analysis were performed using Microsoft Excel (Microsoft Corporation, Redmond, WA). Median waiting time from CT sim to RT initiation before and after intervention were compared descriptively to assess the effectiveness of the QI initiative. Feedback from departmental staff and patients was also documented to assess perceived acceptability and operational impact. This study was conducted as part of an institutional QI program and adhered to ethical standards for QI research.

## Results

At baseline, the median interval from CT sim to the initiation of RT at our institution was 18 days.

**Figure 2.**
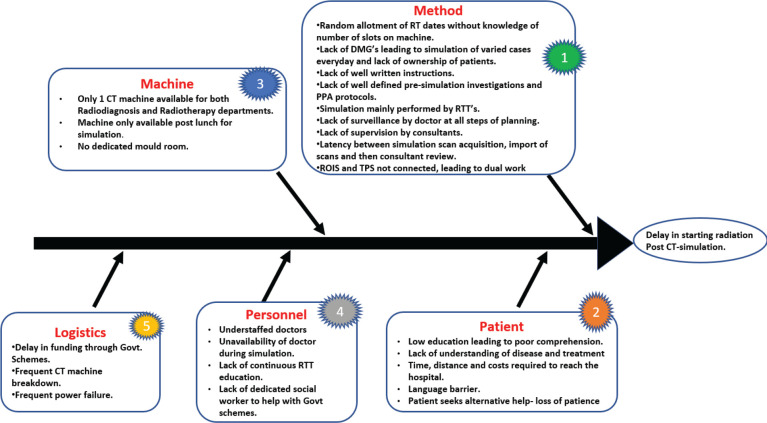
Fishbone analysis categorising systemic causes of delay in RT initiation into five domains. Abbreviations: CT, computed tomography; DMG, Disease Management Group; PPA, Pre Planning Audit; ROIS, radiation oncology information system; RT, radiation therapy; RTT, radiation therapy technologist; TPS, treatment planning system.

As part of the RCA, a Fishbone (Ishikawa) diagram (Figure 2) was constructed to visually organise the contributing factors underlying delays in RT initiation postCT sim. These factors were systematically grouped into five primary domains: personnel (e.g., unclear staff responsibilities), methods (e.g., lack of standardised SOPs), machines (e.g., scan import delays), logistics (e.g., government documentation bottlenecks) and patient-related issues (e.g., inadequate instruction comprehension).

**Figure 3.**
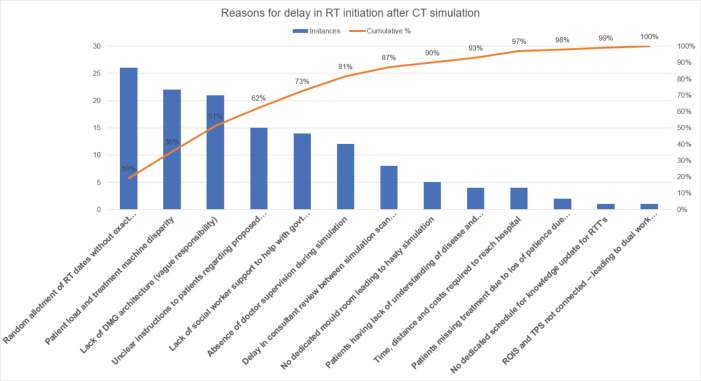
Pareto chart summarising the cumulative percentage of major causes contributing to delay in RT initiation post-simulation. Abbreviations: DMG, Disease Management Group; ROIS, radiation oncology information system; RT, radiation therapy; RTT, radiation therapy technologist; TPS, treatment planning system.

RCA was further supported by Pareto analysis (Figure 3), which identified four major causes of delay:

1. Inefficiencies in RT appointment scheduling
2. Inadequate patient comprehension of preparatory instructions
3. Ambiguity in staff roles and responsibilities

Gaps in coordinated care delivery across services, including delays in processing government documentation, which were addressed through the KEVAT desk, a dedicated patient navigation system designed to streamline access to health schemes.

**Figure 4.**
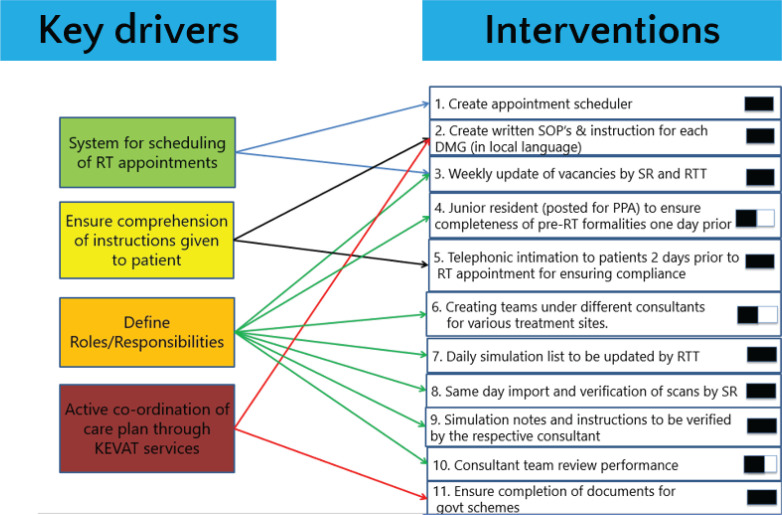
Root causes and interventions for delays in RT initiation. This figure outlines root causes of delay, categorised into four primary drivers and the corresponding corrective interventions implemented. Each color-coded driver (e.g., scheduling, communication, role clarity, KEVAT coordination) is linked to specific actionable steps designed to address the identified bottlenecks. Half and full bars denote the degree of implementation of each intervention (partial versus complete). Abbreviations: DMG, Disease Management Group; PPA, Pre-Planning Audit; RTT, Radiation Therapy Technologist; SOPs, Standard Operating Procedures; SR, Senior Resident.

To address these, a series of targeted, stepwise interventions (Figure 4) were implemented through the PDSA framework. These included:

- Creation of a centralised scheduling system with daily caps on simulation numbers
- Disease-specific SOPs with clearly printed simulation instructions
- Mandatory weekly simulation list updates by RTTs
- Pre-planning audits (PPAs) by junior residents (JRs)
- Daily scan import and verification protocols
- Advance communication with patients regarding logistics and preparation
- On-site completion of financial documentation on the day of simulation
- Consultant-assigned site responsibilities with regular review audits

**Figure 5.**
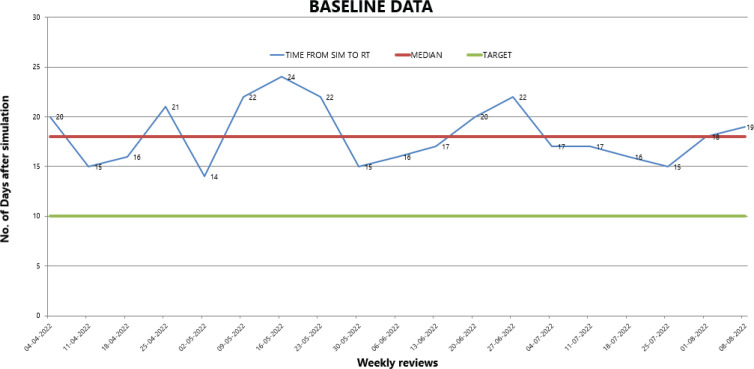
Run Chart displaying the weekly median number of days between CT sim and RT initiation prior to intervention implementation. The blue line represents weekly values, the red line indicates the pre-intervention median (18 days) and the green line marks the target goal of 10 days.

A run chart (Figure 5) was used to plot the weekly RT initiation times across the 9-month period.

**Figure 6.**
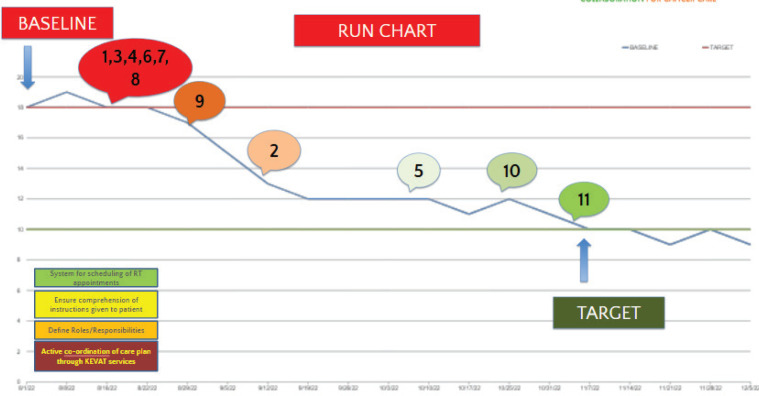
Run chart demonstrating weekly reduction in RT initiation delay with Annotated interventions. The red line marked the baseline average delay of 18 days, while the green line indicated the target of 10 days. Numeric annotations (e.g., 1, 3, 4, 6, 7 and 8) correspond to specific interventions introduced at various times. Larger circles like 9, 2, 5, 10 and 11 highlight critical intervention milestones. The blue trend line shows the progression of delay reduction over time.

A second run chart (Figure 6) tracked weekly median delays from CT sim to RT initiation following the implementation of targeted interventions. Progressive reductions were observed with each cycle. The baseline average delay of 18 days (red line) showed a consistent decline across October and November 2022, corresponding with key interventions such as centralised scheduling (items 1, 3, 4, 6, 7, 8), consultant-led review processes (item 10) and integration of KEVAT documentation on the day of simulation (item 11). These targeted interventions aligned with key driver categories, scheduling, instruction clarity, role definition and coordinated planning, and contributed to achieving the predefined target of 10 days (green line) by mid-November. Weekly metrics fell below the target line in the latter part of the study, indicating durable improvement.

By September 2022, the median time from simulation to RT initiation dropped from 18 to 12 days. Following further process refinements, including consultant performance review meetings and streamlined KEVAT services for the timely completion of documents for government schemes, the median duration was reduced to the target of 10 days by November 2022, representing a 44.4% reduction in waiting time.

In addition to the marked reduction in median simulation to treatment interval, several secondary outcome measures demonstrated significant improvement, underscoring the effectiveness and sustainability of the implemented interventions. Notably, the proportion of patients initiating RT within 10 days of CT sim increased from 30% at baseline to 78% by the end of the study period. This shift reflects not only an improvement in central tendency but also a favourable change in the overall distribution of waiting times, indicating broader systemic impact.

Re-simulation rates, a critical quality indicator reflecting planning inefficiencies and treatment delays, declined from 10% to less than 1%. This reduction was primarily attributable to improved standardisation of simulation protocols, pre-planning audits and timely identification of imaging or positioning issues, which minimised the need for duplicate scans and redundant planning efforts.

Importantly, from October 2022 onward, no patient experienced a delay in RT initiation beyond their clinically indicated start date, reflecting adherence to planned schedules and enhanced operational control.

The revised SOPs were institutionalised in January 2023 and consistently followed over the subsequent 2 years. Departmental audits during this period confirmed that the median CT Sim to treatment interval remained stable within the 10–12 day range, and re-simulation rates were sustained below 2%, compared to 10% at baseline. Additionally, structured quarterly feedback was obtained from clinical staff, including nursing personnel, RTTs, medical physicists and radiation oncology residents. Among 36 anonymised responses collected between March 2023 and December 2024, 89% (*n* = 32) reported no increase in perceived workload, 81% (*n* = 29) noted improved task clarity and 83% (*n* = 30) reported smoother interdepartmental coordination. These findings underscore the long-term operational feasibility and cross disciplinary acceptance of the intervention bundle.

## Discussion

Delays in initiating RT are a well-recognised challenge in oncology; they are frequent, multifactorial and associated with worse clinical outcomes [14]. Even modest postponements may allow for tumour proliferation and disease upstaging, compromising local control and survival, particularly in aggressive malignancies such as head and neck and cervical cancers [15, 16].

In prior work, Xu *et al* [13] employed Failure Mode and Effects Analysis to evaluate vulnerabilities in the RT workflow, particularly in the interval between CT sim and treatment delivery. However, their work remained largely diagnostic. In contrast, our study went beyond risk identification to implement proactive, data-driven interventions aimed at reducing delays. The resulting median CT Sim to treatment interval of 10 days postintervention not only represents an operational success but also carries potential clinical implications in minimising tumour progression during waiting periods.

Delays in treatment initiation are a particularly significant concern in India, where systemic and infrastructural limitations prolong TTI. A recent multicenter study reported a median TTI of 20 days (interquartile range (IQR) 7–39) from diagnosis to therapy, with a median of 27.5 days (IQR 10–49) for RT-specific starts [17]. Against this national benchmark, our reduction of the simulation to treatment gap to 10 days is both substantial and encouraging. It illustrates how targeted workflow improvements can mitigate larger systemic delays. Internationally, high-income countries often enforce stricter timeliness standards.

Rural cancer centers in low and middle-income countries (LMICs) face unique challenges. RT infrastructure in India is often concentrated in urban areas, leading to travel burdens and significant financial and logistical strain for rural patients. These barriers can result in treatment abandonment, missed appointments and worse outcomes. Prior to our intervention, patients frequently waited over 2 weeks after simulation, requiring multiple hospital visits or extended stays, which imposed considerable personal and economic hardship. These challenges align with evidence linking travel burden to poor treatment adherence and survival outcomes among rural cancer patients [18]. Our intervention directly addressed these disparities. A shorter median wait time of 10 days improves the likelihood of patients completing curative RT timely manner, thereby reducing dropout risk and the window for disease progression.

Moreover, shorter waiting periods lower the need for repeat simulations, commonly necessitated by anatomical or tumour changes during prolonged delays. Re-simulations are not only resource intensive but also contribute to additional radiation exposure. One multi clinic study reported a median treatment delay of seven days due to re-simulation and may exacerbate patient anxiety during treatment planning [12]. In our cohort, reduced wait times coincided with lower re-simulation rates, allowing more patients to proceed with the original treatment plan. This is especially critical in resource-limited environments, where scanner availability and staff time are constrained. Avoiding unnecessary re-planning enhances clinical throughput and reduces indirect costs for both institutions and patients.

In addition to improved median wait times, we observed a shift in the entire distribution of waiting periods, with a larger proportion of patients initiating treatment within the desired 10-day timeframe. These improvements mirror results from similar QI initiatives. For example, Simons *et al* [19] achieved a 4-day reduction in median RT wait times (from 20.2 to 16.3 days) via Lean transformation in a Dutch center, with a notable drop in treatment breaches. In their setting, breaches for palliative RT beyond 10 days decreased from 35% to 16%, while breaches of the 28-day limit for curative RT dropped from 17% to 8% [19]. Likewise, Divi *et al* [20] reported success with a structured QI program to expedite adjuvant RT in oral cancer, increasing the proportion of patients initiating radiation within 6 weeks from 62% to 73% and significantly reducing avoidable delays [20].

Despite differing clinical and geographic settings, a common thread across these QI projects is the effectiveness of structured, methodical interventions in improving oncology care timelines. Our postintervention performance demonstrates that RT wait times are not immutable; they can be optimised through deliberate system redesign. The delay drivers may vary, from scheduling inefficiencies to communication lapses, but the diagnostic and improvement methodology remains universally applicable.

Our study leveraged the A3 Lean framework in conjunction with Fishbone analysis, Pareto prioritisation and iterative PDSA cycles. These tools provided a structured pathway for identifying root causes, testing change ideas and monitoring outcomes. The Fishbone diagram helped categorise delay factors across domains like human resources, equipment availability, procedural inefficiencies and patient-related issues. PDSA cycles allowed incremental, evidence-based adaptation of interventions. Staff feedback during PDSA cycles indicated improved workflow clarity and job satisfaction, particularly due to the resolution of previously stress-inducing bottlenecks.

Such agile cycles are widely recommended in healthcare QI efforts for their ability to accommodate local constraints and evolve with feedback. In line with previous work, including that of Divi *et al* [20], who explicitly employed the A3 Lean framework to mitigate delays in surgical to radiation transitions, our methodology proved adaptable and scalable in a rural LMIC setting.

Crucially, the interventions required minimal financial investment and no additional infrastructure. Many LMIC settings suffer not just from a lack of resources but from inefficient utilisation of existing capacity. Our process-oriented approach demonstrates that targeted workflow improvements can bridge some of these gaps. Moreover, this model can be extended across departments, for example, to improve time from biopsy to chemotherapy initiation, or to higher volume institutions with appropriate contextual adaptations.

Finally, reducing CT Sim to treatment intervals is not an isolated operational goal but part of a broader strategy to improve equity in cancer care. Timely initiation of therapy is a recognised quality metric that directly affects patient outcomes. In rural and underserved populations, reducing delays helps mitigate geographic and economic barriers, contributing to improved compliance, better experiences and potentially enhanced clinical outcomes. Our findings show that even in under-resourced contexts, QI methodologies can bring RT performance in line with international standards. This not only supports the mission of equitable cancer care but provides a replicable framework for other LMIC centers striving to optimise oncology workflows.

The sustained implementation of these interventions through 2024 demonstrates strong institutional buy-in and operational resilience. Continued improvements in key metrics, coupled with positive staff feedback across roles, reinforce the feasibility of long-term adoption and replication of this model in other high-volume, resource-constrained oncology setting.

### Strengths

This study is the first to integrate the A3 methodology with complementary QI tools such as Gemba walks, Fishbone analysis, Pareto charts and PDSA cycles to systematically address simulation-to-treatment delays within a rural Indian cancer center. The interventions implemented were practical, resource-sensitive and easily reproducible in similar low-resource settings. Notably, the initiative led to measurable and sustainable improvements in both clinical outcomes, such as reduced waiting times and lower re-simulation rates, and operational performance, including enhanced workflow efficiency and positive staff feedback, underscoring its effectiveness and scalability.

### Limitations

While the interventions yielded measurable improvements in workflow and simulation-to-treatment timelines, long-term patient outcomes such as local control and survival were not assessed and warrant future investigation. Formal statistical testing to compare pre and postintervention data was not performed, which limits causal inference. Also, the introduction of a centralised simulation scheduling system with daily caps may have modestly increased the interval between oncologist recommendation and simulation; however, this parameter was not formally evaluated in the present study. Additionally, patient satisfaction and psychological outcomes such as anxiety were not evaluated using validated assessment tools, representing another area for future QI.

### Future directives

Future efforts should aim to validate the effectiveness and adaptability of QI interventions across a broader range of oncology centers, including high-volume urban institutions, to assess their scalability and generalisability. Also, it should prospectively assess how daily simulation caps influence both recommendation-to-simulation intervals and overall treatment initiation timelines, ensuring continued workflow optimisation. Incorporating formal statistical analyses and longitudinal monitoring of key clinical outcomes, such as survival, local control will enhance causal inference and provide stronger evidence for impact.

Additionally, evaluating patient-centered outcomes is crucial. A structured patient satisfaction survey, particularly focusing on components such as the KEVAT patient navigation services, should be conducted to capture feedback on workflow improvements, patient communication and perceived quality of care. Such feedback will offer valuable insights for iterative refinements and ensure that future interventions remain aligned with patient needs.

By coupling robust evaluation with system-level integration, these steps will help ensure the durability and continued evolution of our QI framework.

## Conclusion

This study demonstrates that delays in initiating RT following CT sim, an often under-recognised barrier to timely cancer care, can be effectively mitigated through a structured, data-driven QI approach, even within the constraints of a rural healthcare setting. By integrating A3 problem-solving methodology with RCA tools (Fishbone and Pareto) and iterative PDSA cycles, we identified critical workflow inefficiencies and implemented resource-appropriate, scalable interventions. Targeted modifications in scheduling practices, role clarity, patient counselling and process checkpoints led to a 44.4% reduction in the median simulation-to-treatment interval (from 18 to 10 days), without adding burden to existing clinical staff. These improvements were accompanied by enhanced workflow efficiency, fewer re-simulations and improved patient adherence.

Importantly, this QI model offers a reproducible and adaptable framework for RT departments in low-resource settings, aiming to optimise treatment timeliness. Long-term sustainability will require continued process monitoring, integration into institutional protocols and periodic re-evaluation to align with evolving clinical demands. Ultimately, timely RT initiation should be viewed not only as an operational goal but as a key determinant of equitable, patient-centered oncology care. Our findings affirm that systemic delays are modifiable and that structured QI initiatives can yield clinically meaningful gains even in resource-limited environments.

## List of abbreviations

A3, A3 Problem Solving Framework (Lean methodology); CTSIM, Computed Tomography Simulation; DMG, Disease Management Group; EQUIP, Enable Quality Improve Patient Care; HNSCC, Head and Neck Squamous Cell Carcinoma; IQR, Interquartile Range; JR, Junior Resident; KEVAT, Patient-navigation and support system of Tata Memorial Centre that assists patients with government schemes, financial documentation and logistical coordination; LMIC, Low and Middle Income Countries; PDSA, Plan-Do-Study-Act; PPA, Pre Planning Audit; QI, Quality Improvement; RCA, Root Cause Analysis; ROIS, Radiation Oncology Information System; RT, radiation therapy; RTT, Radiation Therapy Technologist; SOPs, Standard Operating Procedures; SR, Senior Resident; TPS, Treatment Planning System; TTI, Time to Treatment Initiation.

## Conflicts of interest

The authors declare that they have no conflicts of interest relevant to the content of this article. No financial relationships, commercial affiliations or personal interests influenced the design, execution or reporting of this study.

## Funding

This study did not receive any specific grant from funding agencies in the public, commercial, or not-for-profit sectors.

## Figures and Tables

**Table 1.** EqUIP questionnaire to identify factors contributing to delays in RT initiation post-CT sim.

EQUIP questionnaire: what is leading to delay in RT initiation after CT sim?	
1.	Lack of DMG architecture of patient care (vague responsibility)
2.	Random allotment of dates for RT start leading to CT sim without knowledge of actual machine slot availability
3.	Unclear instructions to patients/relatives about the proposed treatment workflow by the appointment issuing doctor (resident/consultant)
4.	No dedicated mould room leading to hasty simulations and resultant re-simulations
5.	Absence of doctor supervision during most simulations, which are done by RTTs alone (staff crunch)
6.	Delay in consultant review between simulation and planning completeness
7.	ROIS and TPS not connected, leading to dual work and data loss
8.	Patients lacking understanding of disease and treatment
9.	Time, distance and cost barriers to reaching the hospital
10.	Patients missing treatment due to loss of patience resulting from prolonged waiting time
11.	No dedicated scheduling/knowledge update programs for RTTs
12.	Lack of social worker support for assisting patients with government schemes
13.	Patient load and treatment-machine disparity (only one LINAC and Tele-Cobalt unit)

Abbreviations: CT, computed tomography; DMG, Disease Management Group ; LINAC, linear accelerator; ROIS, radiation oncology information system; RT, Radiation Therapy; RTT, Radiation Therapy technologist; TPS, treatment planning system

**Table 2.** Likert scale to score the EqUIP questionnaire.

Strongly disagree	Disagree	Neutral	Agree	Strongly agree
1	2	3	4	5
